# Post-traumatic stress disorder and associated factors among adult war survivors in Northwest Ethiopia: Community-based, cross-sectional study

**DOI:** 10.3389/fpsyt.2023.1083138

**Published:** 2023-04-11

**Authors:** Assefa Agegnehu Teshome, Endeshaw Chekol Abebe, Misganaw Asmamaw Mengstie, Mohammed Abdu Seid, Getachew Yideg Yitbarek, Yalew Melkamu Molla, Nega Dagnaw Baye, Taklo Simeneh Yazie, Gashaw Walle Ayehu, Molla Jemberie Taye

**Affiliations:** ^1^Department of Biomedical Science, College of Health Science, Debre Tabor University, Debre Tabor, Ethiopia; ^2^Department of Paediatrics and Child Health, College of Medicine and Health Science, University of Gondar, Gondar, Ethiopia; ^3^Pharmacology and Toxicology Unit, Department of Pharmacy, College of Health Science, Debre Tabor University, Debre Tabor, Ethiopia; ^4^Department of Human Anatomy, College of Medicine and Health Science, University of Gondar, Gondar, Ethiopia

**Keywords:** PTSD, Nefas Meewcha, prevalence, associated factors, war

## Abstract

**Background:**

A person may endure or witness a traumatic incident, such as being exposed to war, and, as a result, develop post-traumatic stress disorder (PTSD). There is a lack of information about post-traumatic stress disorder in low and middle-income countries such as Ethiopia. However, armed conflict, abuse of human rights, and violence motivated by race are becoming more commonplace. This study aimed to assess the prevalence of PTSD and associated factors among war survivors in Nefas Meewcha Town, South Gondar Zone, Ethiopia, 2022.

**Methods:**

A community based cross-sectional study was carried out. 812 study participants were chosen using a multi-stage sampling process. A face-to-face interview used a post-traumatic stress disorder checklist (PCL-5) to evaluate PTSD. The association between PTSD and other demographic and psychosocial characteristics was investigated using bivariate and multivariable binary logistic regression analysis. A *P*-value of 0.05 was declared as statistical significance.

**Result:**

The prevalence of PTSD in this study was 40.8% with a 95% CI of 36.2 to 46.7. The likelihood of developing PTSD was significantly associated with the fallowing factors. A close family member killed or seriously injured (AOR = 4.53, 95% CI = 3.25–6.46), being female (AOR = 1.98, 95% CI = 1.3–3.0), moderate (AOR = 3.51, 95% CI = 2.52–4.68) and high perceived stress (AOR = 5.23, 95% CI = 3.47–8.26), depression symptoms (AOR = 4.92, 95% CI = 3.57–6.86), anxiety disorder symptoms (AOR = 5.24, 95% CI = 3.72–7.63), a chronic medical illness (AOR = 3.51, 95% CI = 2.52–5.41), physical assault (AOR = 2.12, 95% CI = 1.05–3.72) and being in a war fighting situation (AOR = 1.41, 95% CI = 1.21–3.14).

**Conclusion:**

This study reported that the prevalence of PTSD was high. Being female, having a previous history of chronic medical illness, depressive symptoms, anxiety symptoms, history of a family member or friend was injured or killed, poor social support, high perceived stress, physical assault, and being in a war fighting situation were statistically associated with PTSD. Hence, regular patient assessment by mental health organizations for those with a history of trauma and facilitation of ways to support such residents is highly recommended.

## Introduction

One of the mental diseases connected to exposure to a traumatic or stressful incident is post-traumatic stress disorder (PTSD), which is characterized by intrusion, avoidance, fluctuating mood and cognition, and hyperarousal that lasts for more than a month after the stressful event. Examples of traumatic or stressful events include rape, torture, civil war, vicious physical attacks, accidents, natural catastrophes, murder, threats, kidnapping, the death of loved ones or friends, the loss of a house, and starvation (1, 2). According to the WHO’s Global Disease Burden Survey, mental illness, especially diseases related to stress, will rank as the second-leading cause of disability by the year 2023 (3). Every year, about 8 million adults worldwide have PTSD (4).

The prevalence of PTSD in the general population is becoming higher, particularly in post-conflict and conflict-ridden societies (5). According to studies of the general population, PTSD prevalence ranges between 1 and 5% (6, 7), While it has been shown to range from 3 to 58% in high-risk groups, such as those in conflict areas (7). In 2021, one of the bloodiest civil wars in history in northern Ethiopia drew international attention. The Tigray People’s Liberation Front (TPLF) party and the Ethiopian National Defense Forces (ENDF) were engaged in a civil war (8). Due to this fight, thousands of individuals lost their lives, and many more suffered injuries and traumatic events as they struggled to survive. Additionally, due to violent threats of rape, torture, mutilation, and destruction of property, nearly 2 million people were forced into internally displaced people’s camps (IDPs). Many of them, including children, were kidnaped, making them more vulnerable to psychological disorders, especially post-traumatic stress disorder (PTSD). The TPLF occupied the town of Nefas Meewcha in the Amhara Region between August 12 and August 21, 2021. According to an Amnesty International report, Tigrayan fighters looted medical institutions and private property in addition to mass rapes, gang rapes, physical attacks, and verbal assaults against women (9).

Mental health issues are a significant public health issue on a global scale, accounting for 14% of the total burden of disease (3). A new report from the Norwegian Refugee Council (NRC) and the Internal Displacement Monitoring Center (IDMC) for 2018 estimates that 30.6 million people worldwide were internally displaced due to conflict and disaster, with the majority residing in low-income nations that are occasionally hit by internal conflict and violence. In 2016, nearly 12.6 million people were internally displaced by violence in Africa (10). Studies have revealed severe mental health effects of wars and genocides around the world, impacting both veterans and civilians (11).

According to the results of a meta-analysis, an estimated 242 million adult war survivors living in post-conflict areas were affected by PTSD, while major depressive disorder affects an estimated 238 million adult war survivors (12). The prevalence of PTSD, which adds to the global burden of disease, is estimated to be around 4% worldwide (4). Another systematic review reported that 3–88% of people in the population have PTSD (13, 14). Additionally, a cross-sectional assessment at community levels in Nepal during the conflict time indicated that the prevalence of PTSD was 53.4% (15). Another study on Palestinian refugees in refugee camps during the Al-Aqsa intifada found that post-traumatic stress disorder was reported to be 68.9% (16). In addition, studies conducted among internally displaced people in Nigeria, Africa, revealed that the prevalence of PTSD was 63% (17), in Morocco 19.3% (18), and in South Sudan 28% (19). According to some Ethiopian studies, the Maikadra Massacre victims from the country’s northwest region had a prevalence of 59.8% PTSD (20), and PTSD affects 58.4% of internally displaced people in South Ethiopia (10). Another study’s results from West Ethiopia found that 17.1% of traumatized patients overall had PTSD (21). The prevalence of PTSD was 37.3% in community-based, cross-sectional research on landslide survivors in Addis Abeba, Ethiopia (1). According to a fairly recent community-based, cross-sectional study done in Dessie, Ethiopia, the prevalence of PTSD was 19.4% (22).

Despite the high prevalence of PTSD in conflict areas around the world, only a few studies with highly variable estimates of its prevalence have been reported in Ethiopia, where armed conflict, ethnic violence, and terrorist attacks are on the rise (20, 22). Most of the studies previously conducted rely on self-reporting questionnaires on PTSD symptoms, which gives different prevalence. In addition, it is imperative to do research in this field to provide scientific support for the formulation of prevention and treatment plans for mental health issues during the current and future civilian attacks. Therefore, the purpose of this study is to determine the prevalence of PTSD and associated risk factors among those who have gone through traumatic experiences in the armed conflict-affected town of Nefas Meewcha. The results of this study will assist medical experts, non-profit organizations, and psychological facilities in creating suitable strategies and treatments to deliver evidence-based treatment for PTSD patients.

## Materials and methods

### Study design, period, and settings

A community-based, cross-sectional study was conducted in May 2022 in the town of Nefas Meewcha in northwest Ethiopia. The town is located 741 km from Addis Ababa, 70 km from Debre Tabor, and 175 km from the regional city of Bihar Dar in Ethiopia. According to the Statistical Agency (2011), there are 18,691 people living in the town overall. A total of 9,009 males and 9,682 women (23).

### Study participants and sampling procedure

It employed a multistage sampling approach. The town of Nefas Meewcha has six kebeles (the lowest administrative unit). Using a straightforward random sampling procedure, three kebeles were picked from the full group. Within these three kebeles, participants were then chosen from each kebele using a proportional distribution of the sample size. Then, to choose the study units, a systematic random selection technique was used. Household lists were obtained from the respected health extension workers. The selected households were used to further sort the interview candidates. The next household participant was interviewed if the selected home was closed while data collection was taking place. Seriously, ill people who could not communicate were not allowed. When there were many eligible participants living in a home, one was chosen by lottery. In the study, participants who were at least 18 years old at the time of data collection were included.

### Sample size determination

The maximum sample size was calculated using a single population proportion formula with the assumptions of 59.8% prevalence of PTSD from previous studies in Maikadra, north-west Ethiopia (20). *Z* = 1.96 (standard normal distribution), 95% CI, margin of error of 0.05, and after using a 10% non-response rate, the total sample size was 406. Using design effect two, the final sample size becomes 812.

### Data collection measures

Four skilled clinical nurses used the Amharic version of the questionnaires to conduct in-person interviews using a semi-structured questionnaire to gather data over 1 month. To keep consistency, the questionnaire was created in English, translated into Amharic, and then returned to English. The goal of the study and how to answer any ambiguous questions were covered in training for data collectors. Additionally, they were educated on ethical standards such as obtaining respondents’ informed consent for participation and maintaining confidentiality, anonymity, and data management.

All measures were assessed in a face-to-face interview and the data collector has coded participant’s responses. A 20-item post-traumatic checklist (PCL-5) was used to assess PTSD, with scores ranging from 0 to 80 on a five-point Likert scale (0 = not at all, 1 = a little bit, 2 = moderately, 3 = quite a bit, 4 = extremely). “A score of =33 was used as cut-off for a clinical level of PTSD symptomatology” (24). The PCL-5’s validity and reliability have been examined and demonstrated in several nations, including Iraq (Cronbach’s alpha = 0.85) (25), and Zimbabwe (Cronbach’s alpha = 0.92) (26). The internal consistency (Cronbach alpha) of PCL-5’s in this study was 0.83.

“Social support was measured by the Oslo Social Support Scale (OSSS-3) which ranges from 3 to 14. Those respondents who scored between 3 and 8 were considered to have poor social support, a score of 9–11 was considered to have moderate social support, and a score of 12–14 was considered to have strong social support” (17). The internal consistency (Cronbach alpha) of Oslo-3 social support was 0.79. “Perceived stress was measured using the perceived stress (PSS) scale, which ranges from 0 to 40. Those respondents who scored 0–13 on the PSS were considered to have low perceived stress, a score between 14 and 26 was considered moderate perceived stress; and those who scored 27–40 were considered to have high perceived stress” (7). “Depression was measured by the Patient Health Questionnaire (PHQ-9), with a score of 10 or more suggesting depression” (27). The PHQ-9’s validity and reliability have been evaluated and validated for use in community studies in several countries. Anxiety symptoms were assessed by a subscale adapted from the Depression, Anxiety, and Stress Scale (DASS-21), with a score of eight or more suggesting anxiety symptoms (28). The internal consistency (Cronbach alpha) of (PHQ-9) and Depression, Anxiety, and Stress Scale (DASS-21) were 0.79, and 0.81, respectively. Socio-demographic factors such as sex, marital status, and educational level, as well as psychosocial variables such as chronic medical illness (yes or no), the worst traumatic event (yes or no), and whether family members or friends were injured or killed during the armed conflict (yes or no), were all recorded in the face-to-face interview.

### Statistical analysis

Data was entered into the computer using EPI Info version 7.2 and exported to SPSS version 25 for analysis. Binary logistic regression was used to identify the variables that were related to the dependent variable. Variables with a *P*-value of less than 0.2 in bivariate regression were taken into consideration for multivariate logistic regression. Finally, the adjusted odds ratio (AOR) at the 95% confidence interval was used to evaluate the strength of the association. A *P*-value of 0.05 in multivariate logistic regression was considered statistically significant. Tolerance and variance inflation factors were examined to test for multicollinearity.

## Results

### Socio demographic characteristics of respondents

A total of 804 participants with response rate of 99% were recruited in this study. Of these, 430 (53.5%) were men. The average age of the respondents was 40 ± 12 years old, with a range of 18 to 72 years. Of them, 466 (58.0%) were between the ages of 15 and 40 years old. 614 (76.4%) of the total participants were married. In terms of occupation, 290 (36%) were housewives, and 356 (44.28%) were government employees. Regarding educational status, 330 (41.0%) went to primary school, 140 (17.1%) went to secondary school, and 224 (27.9%) had no formal education (Table 1).

**TABLE 1:** Socio demographic characteristics of study participants at war affected area Nefas Meewcha town, Northwest Ethiopia, 2022 (*n* = 804).

Characteristics		Frequency	Percentage
Age (years)	18−40	466	58.0
	>40	338	42.0
Sex	Male	430	53.5
	Female	374	46.5
Marital status	Married	614	76.4
	Single	130	16.2
	Divorced	20	2.5
	Windowed	40	5.0
Religion	Orthodox	781	97.1
	Others**	23	2.9
Educational status	No formal education	224	27.9
	Primary school	330	41.0
	Secondary school	140	17.4
	Diploma	80	9.9
	Degree and above	30	3.7
Occupational status	Gov. employee	356	44.3
	Daily laborer	56	7.0
	House wife	290	36.1
	Merchant	26	3.2
	Student	66	8.2
	Others*	10	1.2

Others*: retired, NGOs, and farmer, Others**: Muslim, protestant.

### The prevalence of post-traumatic stress disorder

The prevalence of post-traumatic stress disorder among participants was 328 (40.8%; 95% CI = 36.2–46.7), with an estimated prevalence of 32.1 and 50.8% for males and females, respectively, (Table 2).

**TABLE 2:** Proportion post-traumatic stress disorder (PTSD) among the respondents at war affected area of Nefas Meewcha town, Northwest Ethiopia, 2022 (*n* = 804).

Variables	Category	Post-traumatic stress disorder	
		Yes %	No %
Age	18−40	186 (39.9)	280 (60.1)
	>40	142 (42)	196 (58.0)
Sex	Male	138 (32.1)	292 (67.9)
	Female	190 (50.8)	184 (49.2)
Marital status	Married	224 (36.5)	190 (63.5)
	Single	60 (46.2)	70 (53.8)
	Divorced	14 (70)	6 (30.0)
	Windowed	30 (75)	10 (25.0)
Ethnicity	Amhara	328 (40.8)	476 (59.2)
	Others	0	0
Religion	Orthodox	328 (40.8)	476 (59.2)
	Others	0	0
Educational status	No formal education	106 (47.3)	118 (52.7)
	Primary school	114 (35.5)	216 (65.5)
	Secondary school	58 (41.3)	82 (58.7)
	Diploma	38 (47.5)	42 (52.5)
	Degree and above	12 (40)	18 (60)
Occupational status	Government employee	118 (33.1)	238 (66.9)
	Daily laborer	26 (46.4)	30 (53.6)
	House wife	136 (46.9)	144 (53.1)
	Merchant	10 (38.5)	16 (61.5)
	Student	32 (48.5)	34 (51.5)
	Others*	4 (40)	6 (60)
Depression symptoms	Yes	210 (66.5)	106 (33.5)
	No	118 (24.2)	370 (75.8)
Anxiety symptoms	Yes	207 (63.1)	96 (36.9)
	No	121 (24.2)	380 (75.8)
Having chronic medical illness	Yes	89 (72.4)	74 (27.6)
	No	239 (37.3)	402 (67.2)

Others*: retired, NGOs, and farmer.

### Factors associated with PTSD

Post-traumatic stress disorder was significantly associated with being female, being widowed, being a student, being a housewife, having moderate and poor social support, having moderate and high levels of perceived stress, having a family member or close friend injured or killed, being screened positive for depression and anxiety, and having a chronic medical illness.

Multivariable binary logistic regression analysis revealed that PTSD was significantly associated with female sex, single marital status, housewife and being student, having a family member or close friend injured or killed, having poor social support, being screened positive for depression and anxiety symptoms, and having a chronic medical illness. Females had 1.98 times the odds of developing PTSD as males (AOR = 1.98, 95% CI 1.3–3.0). The odds of developing PTSD were 1.5 times higher among singles compared with married ones (AOR = 1.48, 95% CI 1.2–2.5). Having clinical sign of depression (AOR = 5.24, 95% CI = 3.72–7.63) and anxiety (AOR = 5.24, 95% CI = 3.72–7.63) were significantly associated with post-traumatic stress disorder. The odds of PTSD were five-times higher in those with a high perceived stress (AOR = 5.23, 95% CI = 3.47–8.26). The odds of developing PTSD were 4.5 times higher among individuals whose family members or friends were seriously injured or killed (AOR = 4.53; 95% CI = 3.25–6.46). The odds of developing PTSD were 3.5 times higher among individuals who had a chronic medical illness compared with those who had not. Participants who had been directly exposed to physical assault type of traumatic event were 2 times more likely to have PTSD (AOR = 2.12, 95% CI = 1.05–3.72) (Table 3).

**TABLE 3:** Factors associated with post-traumatic stress disorder (PTSD) among the respondents at war affected area of Nefas Meewcha town, Northwest Ethiopia, 2022 (*n* = 804).

Variables		Post-traumatic stress disorder			
		Yes %	No %	COR (95% CI)	AOR (95% CI)
Age	18−40	186 (39.9)	280 (60.1)	1	1
	>40	142 (42)	196 (58)	1.1 (0.7–1.6)	1.1 (0.7–1.6)
Sex	Male	138 (32.1)	292 (67.9)	1	1
	Female	190 (50.8)	184 (49.2)	2.2 (1.5–3.3)**	1.98 (1.3–3.0)**
Marital status	Married	224 (36.5)	390 (63.5)	1	1
	Single	60 (46.2)	70 (53.8)	1.5 (0.9–2.6)	1.48 (1.2–2.5)*
Divorced	14 (70)	6 (30)	4.1 (1.0–16.0)*	1.38 (0.8–2.5)
Windowed	30 (75)	10 (25)	5.2 (1.9–14.8)*	2.6 (0.6–11.2)
Educational status	No formal education	106 (47.3)	118 (52.7)	1	1
	Primary school	114 (35.5)	216 (65.5)	-0.59 (0.4–0.9)	-0.6 (0.4–0.9)*
	Secondary school	58 (41.3)	82 (58.7)	-0.79 (0.4–1.4)	-0.8 (0.4–1.5)
	Diploma	38 (47.5)	42 (52.5)	1.0 (0.5–2.1)	1.0 (0.5–2.2)
	Degree and above	12 (40)	18 (60)	-0.74 (0.3–2.2)	-0.8 (0.3–2.23
Occupational status	Government employee	118 (33.1)	238 (66.9)	1	1
	Daily laborer	26 (46.4)	30 (53.6)	1.7 (0.8–3.9)	1.7 (0.8–3.9)
	House wife	136 (46.9)	144 (53.1)	1.8 (1.1–2.8)**	1.9 (1.1–2.8)*
	Merchant	10 (38.5)	16 (61.5)	1.3 (0.4–4.0)	1.3 (0.4–4.0)
	Student	32 (48.5)	34 (51.5)	2.2 (1.1–4.7)**	2.1 (1.0–4.50)*
	Others*	4 (40)	6 (60)	1.2 (0.2–7.7)	1.3 (0.2–8.3)
Social support	Poor social support	231 (44.2)	292 (55.8)	1.88 (1.19–3.23)**	1.47 (1.15–3.11)**
	Moderate social support	76 (36.2)	134 (63.8)	1.35 (0.75–2.42)	1.31 (1.26–1.43)*
	Strong social support	21 (9.1)	50 (90.9)	1	1
Worst events	Physical assault	177 (47.2)	198 (52.8)	2.32 (1.09–4.95)**	2.12 (1.05–3.72**
	Being in a war fighting situation	82 (36.9)	140 (63.1)	1.52 (0.79–3.32)	1.41 (1.21–3.14)*
	Destruction of personal property	59 (34.5)	112 (65.5)	1.37 (0.62–3.03)	1.29 (0.57–2.36)
	Did not access medical care during the event	10 (27.8)	26 (15.2)	1	1
A family member or friend was injured or killed	Yes	84 (74.3)	29 (25.7)	5.31 (3.38–8.32)**	4.53 (3.25–6.46)**
	No	244 (35.3)	447 (64.7)	1	1
Perceived stress	Low perceived stress	29 (15.8)	155 (84.2)	1	1
	Moderate perceived stress	184 (44.3)	231 (55.7)	4.26 (2.74–6.62)*	3.51 (2.52–4.68)*
	High perceived stress	115 (56.1)	90 (43.9)	6.83 (4.21–11.07)**	5.23 (3.47–8.26)**
Clinical depression	Yes	210 (66.5)	106 (33.5)	6.39 (4.68–8.72)**	4.92 (3.57–6.86)**
	No	118 (24.2)	370 (75.8)	1	1
Clinical anxiety	Yes	207 (63.1)	96 (36.9)	6.77 (4.93–9.39)**	5.24 (3.72–7.63)**
	No	121 (24.2)	380 (75.8)	1	1
Having chronic medical illness	Yes	89 (72.4)	34 (27.6)	4.40 (2.87–6.74)**	3.51 (2.52–5.41)**
	No	239 (37.3)	402 (67.2)	1	1

Others* retired, NGOs, and farmer,*P < 0.05, **P < 0.01.

## Discussion

War is complicated and may leave populations permanently traumatized. PTSD is the most common psychopathology and a serious public health concern after trauma or disaster (29). Populations affected by conflict are more likely to experience psychological discomfort and a variety of mental health issues (30). This population-based study aimed to assess the prevalence of PTSD in northwest Ethiopia following the civil war. The study’s findings indicated that PTSD was more common in the studied communities. Additionally, due to the high prevalence of PTSD in the study areas, well-coordinated public mental health measures must be launched. In order to improve people’s ability to function regularly and contribute to society, it is crucial to lessen the prevalence of mental diseases, notably post-traumatic stress disorder (PTSD) (31).

The results of the current study showed that among war survivors with a 95% CI of 36.2–46.7%, the prevalence of PTSD was 40.8%. This result was in line with those from research done in Gonder and Felege-Hiwot compressive specialized hospitals (37.2%) (32), in Addis Ababa, Ethiopia, Koshe Land Slide Survivors (37.3%) (1), and in Jewish Holocaust survivors (39%) (33). However, the prevalence of PTSD in the current study was lower than that reported in Keneya, which reported a prevalence rate of PTSD of 60% (34), 63% in North Eastern Nigeria (35), 59.8% in Maikadra town, North West Ethiopia (20), 58.4% among internally displaced individuals in Ethiopia (10) and 52.9% in Kenya (36). The variability in level of PTSD observed among the different African countries may result from the type of trauma and durations experienced by the study population (greater exposure to multiple types of traumatic events may expose greater PTSD severity).

In contrast, this study found a greater prevalence of PTSD than the studies carried out in Somalia, Jigiga (16.3%) (37), and in South Asia (7.27%) (38). The length of the vulnerable period, the degree of exposure to traumatic events, the tools and cut-off points used to evaluate PTSD, and the study design may all play a role in the discrepancy. Other variances could result from how data was collected and how studies were carried out in the aftermath of the attack.

Regarding the associated factors of physical assault (2.12 times), poor social support (1.47 times), being a housewife (1.9 times), and being single (1.48 times), they were more likely to develop PTSD. This study revealed that the odds of developing PTSD were 1.98 times higher in females compared to males. This report was comparable to studies done in south Ethiopiain Addis Ababa, Ethiopia (1), and in Mikadra, Ethiopia (20). This could be because women are more likely than men to experience psycho-trauma due to a lower threshold, or it could be a direct psychological effect of rape or other sexual abuse (39, 40). When compared to married people, the odds of developing PTSD were 3.6 times higher among widowed individuals. This report was consistent with studies on PTSD among UK military personnel (41).

Study participants whose close friends or family members suffered major injuries during the war had 4.5 times higher odds of developing PTSD. This report was consistent with previous studies of the Maikadra Massacre (20), the Rwandan Genocide (42), and the Peru earthquake (43). The odds of developing PTSD among individuals who had moderate and severe perceived threats to life were 3.5 and 5.2 times greater, respectively, which is consistent with another Ethiopian study (20). The odds of developing PTSD among those with symptoms of depression and anxiety were five times higher as compared to their counterparts. This is consistent with research on veterans held in the King County Jail system in Seattle, King County, Washington, and Kenya (44–46). This might be a result of the fact that participants with depressive and anxiety symptoms are more likely to have PTSD than respondents without depressive and anxiety symptoms (35).

Finally yet importantly, we discovered that participants who had a history of chronic medical conditions were 3.5 times more likely to experience PTSD than their counterparts. Similar to what has been discovered in previous studies from Kenya (47), South Korea (48). Participants with a history of a chronic medical condition may exhibit greater neurochemical imbalance and neuronal damage than participants without such a history. As a result, when exposed to stressful conditions, people with neuron-chemical imbalance caused by a history of chronic illness develop PTSD more quickly (35).

## Strengths and limitations

We used a sizable sample of the population, and the findings may be helpful to nations in the area devastated by the war. Due to the cross-sectional nature of the study, we were not able to verify whether the depressive and anxiety symptoms, as well as substance use, preceded or followed the PTSD. We did not use a structured clinical interview to ascertain the diagnosis of PTSD. Furthermore, this is a community survey; sampling was not intended to be representative according to relevant characteristics. Another limitation was the lack of previously validated Amharic versions of questioners.

## Conclusion

In this study, the prevalence of PTSD was found to be high (40.8%). Having a family member or close friend injured or killed, having poor social support, being female, being screened positive for depression and anxiety, and having a chronic medical illness were all associated with PTSD with a *p*-value less than 0.05. The concerned organization may then practice techniques that can be used for early detection, prevention, and intervention to reduce the incidence of PTSD in war survivors.

## Data availability statement

The raw data supporting the conclusion of this article will be made available by the authors, without undue reservation.

## Ethics statement

The studies involving human participants were reviewed and approved by Health Science College Ethics Committee of Debre Tabor University. The patients/participants provided their written informed consent to participate in this study.

## Author contributions

AAT contributed to study conception and design, data analysis, interpretation, and manuscript drafting. ECA, MAM, TSY, MJT, MAS, and NDB contributed to the conceptual idea of the manuscript. GWA, GYY, and YMM contributed in drafting the objectives and wrote the manuscript. All authors were involved in reviewing the final draft of the manuscript, revision of the manuscript, and approved the submitted version.
